# Technique for Determining Fluids Motion Characteristics in the Vicinity of Ferromagnetic Solids Under Magneto-Chemical Treatment

**DOI:** 10.1186/s11671-015-1150-6

**Published:** 2015-11-14

**Authors:** Dmytro O. Derecha, Yury B. Skirta, Igor V. Gerasimchuk

**Affiliations:** Institute of Magnetism, National Academy of Sciences of Ukraine and Ministry of Education and Science of Ukraine, Vernadsky Blvd. 36b, 03142 Kyiv, Ukraine; National Technical University of Ukraine “Kyiv Polytechnic Institute”, Peremohy Ave. 37, 03056 Kyiv, Ukraine

**Keywords:** Electrochemical reaction, Electrolyte, Ferromagnetic surface, Corrosion, Metallic compounds, 82.40.Ck, 47.65.-d, 82.20.-w

## Abstract

We report on new technique for determining and visualization of fluids motion in the vicinity of magnetized ferromagnetic surfaces under chemical dissolution and autocatalytic formation of spatiotemporal structures. The proposed method of obtaining data on the motion of fluids does not require the use of additional inclusions and allows to avoid the distortions caused by such inclusions or other changes in the nature of the reaction. The developed technique allows to obtain both the integral dependences and time frequencies distributions of the fluids over the volume of the medium under investigation.

## Background

The interaction of metal surfaces with electrolytes under the influence of an external magnetic field and the autocatalytic formation of spatiotemporal structures, such as, for example, Belousov-Zhabotinsky reaction [1, 2] and Rayleigh-Bénard convection [3–5], is of great interest in the recent years. The classical examples of such structures are the wave propagation along magnetized surfaces, the hydrodynamic mixing of electrolyte during their electrochemical treatment, etc. [6, 7]. The formation of such structures is caused by the nonlinearities in the reaction mechanism, such as catalysis or phase transitions on the surface.

The determining dynamic characteristics of fluids motion is of great fundamental and applied interest because of timeliness of self-organization processes, formation of dissipative structures, and changing in the kinetics of the reaction [8–10] in physical and chemical interactions of magnetized surfaces with electrolytes. Such systems are relatively simple model objects for investigations of the processes at the metal-electrolyte interfaces and, thus, widely used in approbations of theoretical models. The magnetic field in this case plays the role of an additional parameter that allows to modify existing structures or create new ones by changing or substitution of reaction mechanisms [11].

The measurements of the dynamic characteristics of the motion of fluids are related with the need of entering marker particles in the medium under investigation or usage other mechanical methods [12]. Such methods and the usage of additional inclusions may cause the distortions in the nature of reaction. This leads to decreasing in the accuracy of measurements.

In our previous paper [13], we proposed the method for local measurement of the frequency characteristics of electrolytes motion. In order to obtain the dynamic picture of the behavior of fluids that interact with metal surfaces over the volume of the medium in the vicinity of magnetized surfaces, we modified the proposed method by changing the geometry of filming and radiation source. The usage of the near-infrared scattered radiation allowed increasing the contrast of the resulting image due to a higher absorption of radiation by a medium under investigation. The developed method allows us to obtain the distributions of characteristic frequencies of fluids both over the volume and in the selected area of the medium under investigation.

## Methods

**Fig. 1:**
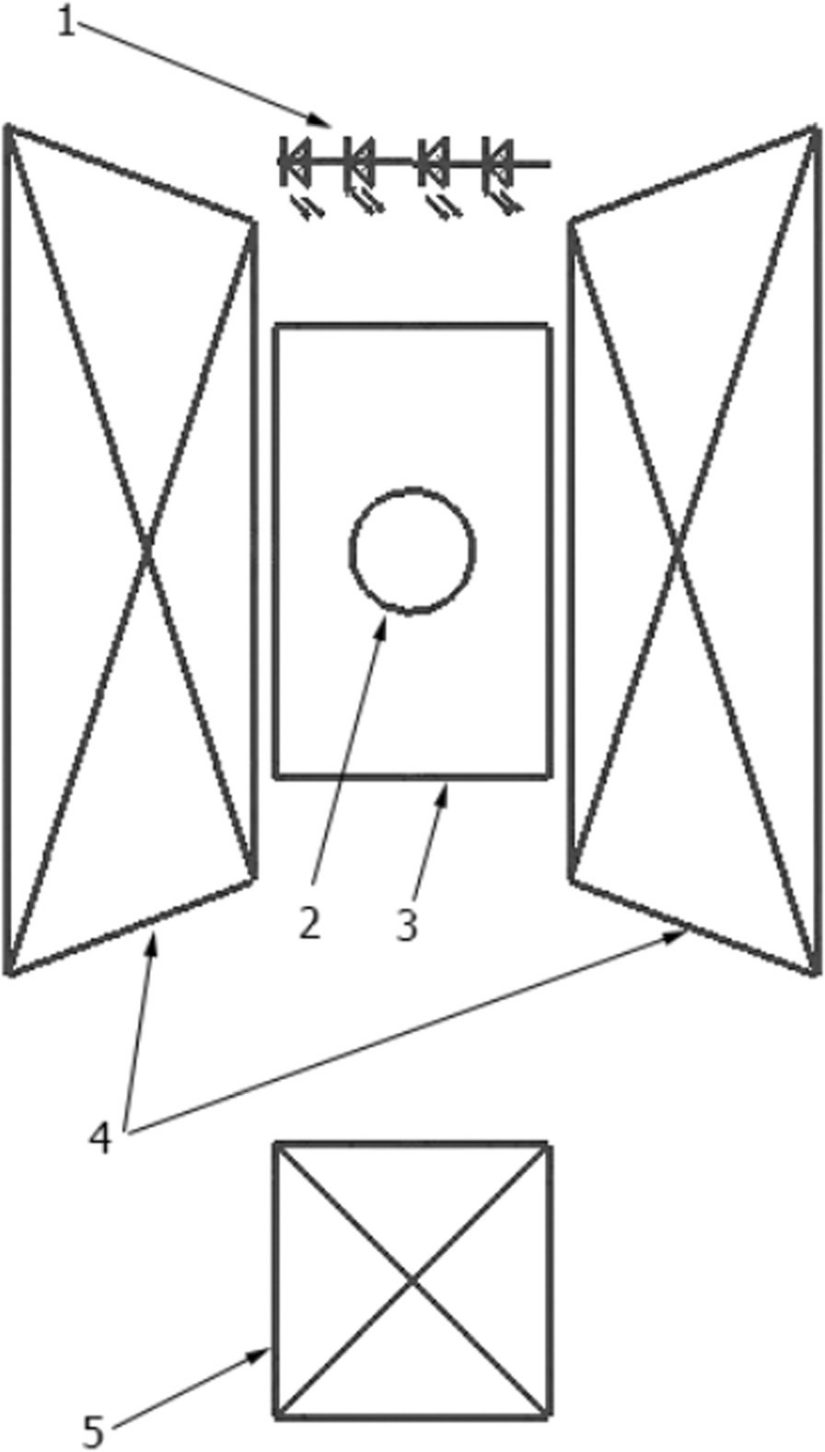
Experimental setup. *1*—LED assembly, *2*—sample, *3*—electrochemical cell, *4*—pole tips of electromagnet, and *5*—camera

In order to obtain an overall picture of the dynamic behavior of fluids that interact with metal surfaces under the influence of an external magnetic field, the method described previously in [13] was modified by changing the radiation source and geometry of filming (see Fig. 1). The electrolyte solution was illuminated by the LED operating in near-infrared scattered radiation of 850 nm wavelength that allowed to increase the resulting contrast of recording due to a higher radiation absorption by a medium under investigation. It should be noted that the resulting contrast under illumination of 850 nm near-infrared radiation is much higher than that at lower frequencies.

Gorobets et al. have shown in [14–16] that the corrosion of the ferromagnetic ball in an external uniform magnetic field is accompanied by arising of sphere-like electrolyte flows in the regions with maximal corrosion activity, i.e., with maximal magnetization. Due to these fluxes, the refractive index and the optical clarity of the medium are changed.

For the current investigation, we used the steel ball (AISI 52100 Alloy Steel, manufactured according to GOST 801-78 [17]) mounted on a nonconductive holder and placed in a quartz electrochemical cell filled with a 7 % solution of nitric acid and the external magnetic field of 0.16 T. This corresponds to the experiment in [13]. The image was recorded by CELESTRON NexImage 5 camera in the mode of 52 frames per second. The frame size was 640 by 480 pixels. The most strong and regular image changes were observed in a very close vicinity of the ball in the regions with maximal magnetization. The movement of liquid in the cell was traced by camera along with the changes of radiation absorption.

**Fig. 2:**
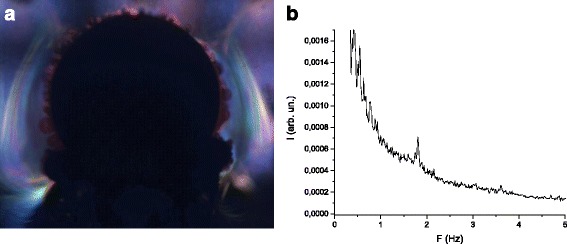
Visualization (**a**) and frequency distribution (**b**) of the medium motion for the whole duration of filming

**Fig. 3:**
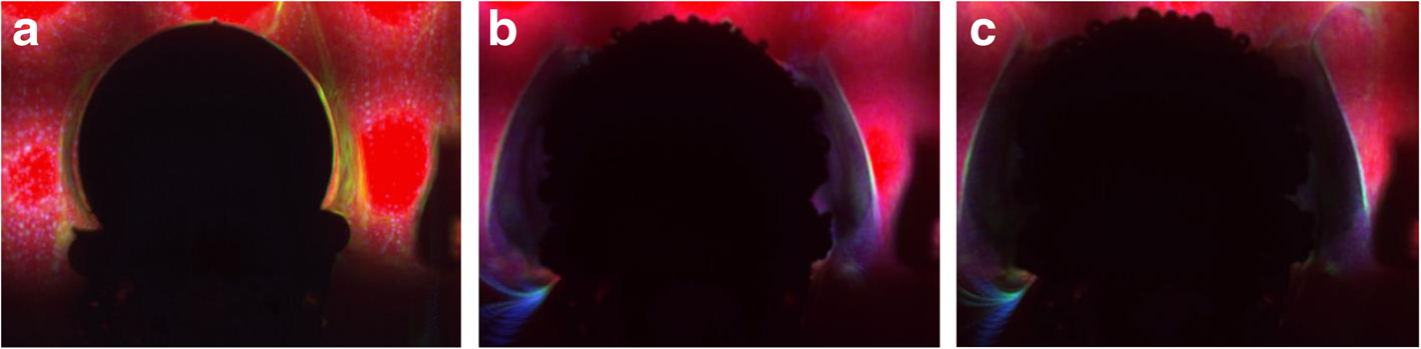
Visualization results and frequencies characteristics of the medium motion for time intervals: **a** 0–12 s, **b** 144–156 s, and **c** 288–300 s

The treatment of the obtained data was carried out in accordance with the algorithms described in details in [13]. An important feature of the present study to compare with [13] is that the Fourier transform was performed by using the floating window length. This allowed to determine the dynamic characteristics of the medium motion both for the whole duration of filming (Fig. 2) and for the time intervals corresponded to the window lengths (Fig. 3).

The second advantage of the present method is that we obtain the integral and time frequencies distributions of the medium motion both over the whole volume and in the selected area of the medium under investigation.

Note that the phase dependences were not analyzed because between the oscillations of different pixels, a significant phase shift was observed, and the frequency distribution map was built on the illuminated area. For each individual pixel, the dependence of its intensity on time was obtained, and further, the Fourier transform was applied to each such dependence. Three regions, from 0 Hz till 0.5 Hz, from 0.5 Hz till 2.0 Hz, and from 2.0 Hz till 15.0 Hz, were allocated in the frequency dependences. The boundaries were chosen conventionally, i.e., below the main maximum peak, and the maximum itself was chosen above the maximum value. As a result, three images were obtained, and each pixel was proportional to the resultant frequency intensity in the specified borders. At the same time, the normalization for each image was performed separately, i.e., the maximum intensity within the specified range corresponds to the maximum brightness.

## Results and Discussion

Let us discuss the obtained frequency characteristics of the processed image. As we can see from Fig. 2b, the maximum peaks of the characteristic frequencies are clearly distinguished on the background of noise. The most intensive are the peaks at frequencies 0.88 and 1.7 Hz which correspond to the frequencies of the most notable changes in the optical characteristics of the solution. The steady component is not shown on the graph in order to improve the perception. The comparison of the obtained data with the theoretical and experimental studies [13–16, 18, 19] shows that the results are in the range of the characteristic frequencies of the magnetohydrodynamic (MHD) electrolyte mixing. The maximum of 1.7 Hz is much lower than the first one of 0.88 Hz and perhaps is the second harmonic. The second harmonic in this case can be generated due to nonuniformity of the rotational motion of electrolyte and to the nonlinearity of the camera CCD matrix.

At high signal intensity, the CCD matrix enters the saturation regime and works with large distortions that lead to a big steady component and the appearance of higher harmonics. The background signal is mostly composed of low frequency noises. This means it is necessary to carefully choose the intensity of a laser beam and the area of the image analysis. The area should be bright enough to provide a sufficient signal-noise ratio, but does not expose the matrix which must operate in the linear mode.

## Conclusions

Finally, the conducted investigations and the developed methods of the results processing show the possibility of determining the dynamic characteristics of the rotational motion of electrolytes during electrochemical reactions in an external magnetic field. The developed technique allows to determine the characteristic frequencies of fluids motion without introducing the marker particles, i.e., without the changing of the electrolyte structure. The obtained results show that under the chosen experimental conditions, the typical frequencies of the electrolyte motion are 0.88 and 1.70 Hz that is in good agreement with the theoretical data. Furthermore, in the case of video recording of the whole cuvette in a wide laser beam, most likely, we can simultaneously obtain the data on the rotational motion of the electrolyte for different distances from the electrode surface and build the corresponding image. In general, the developed technique, with certain modifications, can be used for the study of frequency characteristics of the motion of fluids, gases, and small objects without a direct influence on the medium under investigation.
